# Supplementary material for: Local prediction-learning in high-dimensional spaces enables neural networks to plan
Source: Nat Commun. 2024 Mar 15;15:2344. doi: 10.1038/s41467-024-46586-0 (PMC10943103; doi:10.1038/s41467-024-46586-0)
Supplement: Supplementary file 3 — Description of Additional Supplementary Files [file 41467_2024_46586_MOESM3_ESM.pdf]

Description of Additional Supplementary Files

Supplementary Movie legends

Supplementary Movie 1: PCA analysis of the emergence of a cognitive map in the rectangular 2D grid environment: During the learning phase, the Cognitive Map Learner (CML) successfully formed a perfectly symmetric 2D map, representing the spatial relations among the observations encountered during learning. This was achieved despite the fact that not all edges of the graph were encountered.

Supplementary Movie 2: Random motor movement: During the learning phase the CML was able to explore the environment by executing random actions and observing how they change the environment (motor babbling). One sample learning trajectory of such random movements can be seen in this video.

Supplementary Movie 3: Ant goes to a target: In the planning phase, the CML can be used to move the ant to any given target location. In this video, the ant is tasked to move to a specific location, which is 20 meters away and marked by the cylinder. The target observation given to the CML every time step is the current observation but where the coordinates are set to the coordinates of the target location.

Supplementary Movie 4: Ant flees from predator: The CML can also handle dynamic targets, which change every time step. In this video the task for the CML is to flee from a predator, which has been visualized by a big cube. The target observation given to the CML every time step is the current observation but where the coordinates are set to a location pointing straight away from the predator. The video illustrates this scene in bird's-eye view.

Supplementary Movie 5: Ant chases some prey: In this video the CML is requested to control the ant such that the ant catches a moving prey object. The prey object is visualized as a small red sphere. The target observation given to the CML every time step is the current observation but where the coordinates are set to the coordinates of the prey. The video illustrates this scene in bird's-eye view.
